# Seed Priming with Glutamic-Acid-Functionalized Iron Nanoparticles Modulating Response of *Vigna radiata* (L.) R. Wilczek (Mung Bean) to Induce Osmotic Stress

**DOI:** 10.3390/mi14040736

**Published:** 2023-03-26

**Authors:** Tauheed Ul Haq, Rehman Ullah, Muhammad Nauman Khan, Moona Nazish, Saeedah Musaed Almutairi, Rabab Ahmed Rasheed

**Affiliations:** 1 Department of Botany, University of Peshawar, Peshawar 25120, Pakistan; 2 Department of Botany, Islamia College Peshawar, Peshawar 25120, Pakistan; 3 University Public School (UPS), University of Peshawar, Peshawar 25120, Pakistan; 4 Department of Botany and Biodiversity Research, University of Vienna, 1010 Vienna, Austria; 5 Department of Botany and Microbiology, College of Science, King Saud University, P.O. 2455, Riyadh 11451, Saudi Arabia; 6 Histology & Cell Biology Department, Faculty of Medicine, King Salman International University, South Sinai 11341, Egypt

**Keywords:** Glu-FeNPs, inorganic fertilizers, seed priming, osmotic stress, mung bean

## Abstract

Rising soil salinity is a major concern for agricultural production worldwide, particularly in arid and semi-arid regions. To improve salt tolerance and the productivity of economic crop plants in the face of future climatic changes, plant-based solutions are required to feed the continuously increasing world population. In the present study, we aimed to ascertain the impact of Glutamic-acid-functionalized iron nanoparticles (Glu-FeNPs) on two varieties (NM-92 and AZRI-2006) of mung beans with different concentrations (0, 40 mM, 60 mM, and 80 mM) of osmotic stress. The result of the study showed that vegetative growth parameters such as root and shoot length, fresh and dry biomass, moisture contents, leaf area, and the number of pods per plant were significantly decreased with osmotic stress. Similarly, biochemicals such as protein, chlorophylls, and carotenes contents also significantly declined under induced osmotic stress. The application of Glu-FeNPs significantly (*p* ≤ 0.05) restored both the vegetative growth parameters and biochemical contents of plants under osmotic stress. The pre-sowing treatment of seeds with Glu-FeNPs significantly ameliorated the tolerance level of *Vigna radiata* to osmotic stress by optimizing the level of antioxidant enzymes and osmolytes such as superoxide dismutase (SOD), peroxidase (POD), and proline contents. Our finding indicates that Glu-FeNPs significantly restore the growth of plants under osmotic stress via enhancing photosynthetic activity and triggering the antioxidation system of both varieties.

## 1. Introduction

Soil salinity is a major abiotic stressor, particularly in arid and semi-arid regions of the world. It is estimated that more than half of all global cropland will be salinized by 2050 due to increasing salinity [1]. Likewise, there are 4.5 million hectares of salt-affected land in Pakistan. Economically, Pakistan is primarily dependent on agriculture [2], and nearly 20% of the national income is generated by this sector. Salinity significantly decreases seed germination, seedling growth, photo-assimilates, and other vital physiological processes [3]. Salinity affects the growth of the plant by reducing osmotic potential, specific ion toxicity, and nutrient availability [4], causing the production of ROS such as anions of superoxide and also resulting in anabolic and catabolic toxicity in the chloroplast and mitochondria, which are considered the most important organelles of the cell [5]. The processes of photosynthesis and leaf area are reduced under salinity stress in relation to stomatal closure and assimilation of CO_2_ by reducing the activity of RUBISCO [6].

Meanwhile, the world population is emerging rapidly and it is estimated that by 2025, the figure will reach eight billion. This figure is more likely to cross nine billion by the year 2050. As a result, the cultivated land is decreasing rapidly and negatively affecting the agricultural productivity. The irrigation process and the use of chemical fertilizers are very important for crops to obtain maximum and quality yield. However, the indiscriminate utilization of organic fertilizers leads to many health problems and also causes environmental pollution [7]. Furthermore, the use of organic fertilizers also severely affects the underground water table and results in eutrophication in different ecosystems [8]. To solve the issues of the high salinity, maximum cost, adverse effects of fertilizers, rapidly increasing population of the world, and inaccessibility of agricultural land, innovation in agricultural science is one of the mainstays of enhancing farm productivity [9].

Nanotechnology presents a solution for environmental, health, and technological challenges, including agricultural science [10,11,12]. Nanotechnology has a great ability to save plants from harm, diagnose plant and animal diseases, improve food quality, and reduce environmental pollution [13,14]. The importance of this technology in agricultural science is quite recent compared to other fields [15,16]. For valuable and effective crop production, the utilization of nanoparticles as nanofertilizers is one of the major roles in effective crop production. These nanofertilizers significantly improve plant growth, crop yield, and plant tolerance when used in a suitable concentration [17]. The application of metallic nanoparticles (Zn-NPs, Cu-NPs, Fe-NPs, etc.) significantly enhances plant growth and improves the photosynthetic rate and seedling growth in *Elodea desaplanch*. It is also reported that silicon nanoparticles positively affect the growth of the basil plant under salt stress [18]. The foliar application of TiO2-NPs at a 10 mg/L concentration enhances the length of root, length of shoot, chlorophyll, protein, and antioxidant enzymes in mung beans [19].

Mung bean (*Vigna radiata* L), family Fabaceae, is considered the most useful and widely utilized pulse crops all over the world [20]. Mung bean has high nutritional value as the seeds are rich in proteins, fibers, amino acids, fats, and carbohydrates. It also contains balanced contents of calcium, phosphorous, and polyphenols [21]. For 2000 years, the people of China have used mung bean as a typical food as it has some valuable properties such as lowering stroke heat, minimizing gastric disorders, and also having antidiabetic, anti-inflammatory, and anticancer potentials [22]. The production of mung bean is decreasing day by day all over the world due to salinity, overpopulation, and the imbalanced use of chemical fertilizers. So, it is essential to advance the poor production of Mung bean.

The research data show that iron is an important microelement and has a significant role in many metabolic processes of plants. Similarly, glutamic acid also improves the rate of photosynthesis, the potential of plant nitrogen uptake, and soil nitrogen availability, promoting the nutrient absorption ability of plants [23]. Fe-NPs improve leaf weight, leaf area index, and the number of plants per plant [24], enhancing the protein content, Fe concentration in grain, grain yield, and straw of wheat [25]. It is reported that the application of iron nanocrystals to *Helianthus annus* minimized the unfavorable effects of abiotic stresses, improved the absorption of NKP, and enhanced the plant growth and yield significantly [26]. The application of iron nanoparticles significantly increased the essential oil amount in *Mentha piperita* compared to the control [27].

The current work aimed to determine the beneficial potential of glutamic-acid-functionalized iron nanoparticles (Glu-FeNPs) on the physico-biochemical parameters of two varieties (NM-92 and AZRI-2006) of mung bean under varying concentrations of NaCl-based osmotic stresses.

## 2. Methods and Materials

### 2.1. Synthesis of Glutamic-Acid-Capped Iron Nanoparticles (Glu-FeNPs)

Glutamic-acid-capped iron nanoparticles (Glu-FeNPs) were synthesized by following the ion reduction method where aqueous Fe^+2^ (Iron II salt) in an alkaline medium (pH 11) was reduced by a tannic acid solution with continuous stirring. The resulting Fe-NPs suspension was centrifuged, washed three times, and again dispersed in 1 mM aqueous solution of glutamic acid under vigorous stirring. The resulting Glu-FeNPs were centrifuged, washed with ethanol followed by distal water to removed unreacted materials, dried, and stored in closed vials under ambient conditions [26].

### 2.2. Characterization of Nanoparticles

To determine the morphological features of the Glu-FeNPs through a scanning electron microscope (SEM), the dry sample of Glu-FeNPs was subjected to FE-SEM (JSM-5910-JEOL-JAPAN) at CRL, University of Peshawar. Similarly, for the elemental composition, the oven-dried (50 °C) Glu-FeNPs’ powdered form was utilized for energy-dispersive X-ray spectroscopy (EDX) analysis using the Oxford Inca 200 SEM instrument equipped with a Thermo EDX attachment (CRL, University of Peshawar, Peshawar, Pakistan). The X-ray diffraction (XRD) pattern of Glu-FeNPs was recorded by nickel monochromatic filtering with Cu-Kα radiation (λ = 1.5406 Å), using a JEOL JDX 3532 X-ray diffractometer (CRL, University of Peshawar). For determining functional groups associated with glutamic acid in Glu-FeNPs, infrared spectroscopy was carried out within the range from 4000 to 400 cm^−1^ at 4 cm^−1^ resolutions (PerkinElmer C94012, Waltham, Massachusetts, USA).

### 2.3. Lab Experimental Details

Seeds of *Vigna radiata* were surface-sterilized for 10 min in a 5% NaClO solution, washed with distal water, dried, and primed in a suspension of Glu-FeNPs (150 mg/L) for 5 h. The primed seeds were kept on double folds of filter paper in a Petri plate with different concentrations of NaCl-based osmotic stresses (0, 40 mM, 60 mM, and 80 mM of NaCl), and incubated at 25 °C. Each treatment was replicated three times, with ten seeds in each. The seeds primed with 150 mg/L of Glu-FeNPs had the most significant response under the various levels of osmotic stress selected for the field experiment. The data for germination rate and seedling growth (plumule length and radical length) were obtained.

### 2.4. Pot Experiment Details

The experimental work was carried out in a completely randomized block design with triplicates in the greenhouse under natural conditions of temperature (27–38 °C), light, and humidity (30%) in the Department of Botany University of Peshawar, Pakistan (34.0086° N, 71.4878° E). The sandy-loam soil was used for cultivation with pH 6.875 and EC 0.273 dsm^−1^. The experimental units were divided into four blocks based on levels of salinity (0, 40 mM, 60 mM, and 80 mM NaCl), and the primed seeds (150 mg/L of Glu-FeNPs) of both varieties (NM-92 and AZRI-2006) were sowed in pots (15 cm height and 10 cm diameter) filled with 2 kg of soil. The seeds were divided into the following groups according to the primed solution for each variety, as shown in Table 1.

### 2.5. Measurement of the Vegetative and Yield Parameters

After seven weeks, the plant was harvested, and the vegetative growth parameters such as the length of roots and shoots, leaf area, fresh and dry biomass, and number of pods/plant were measured.

### 2.6. Measurement of Biochemical Parameters

An amount of 0.5 g of fresh leaves was homogenized in 10 mL of deionized water to determine the total soluble sugar contents and centrifuged (4000 rpm) for 15 min, and 0.1 mL of supernatant, 80% phenol, and 5 mL of conc. H_2_SO_4_ were added. The optical density (OD) of the reaction mixture was noted at 420 nm with the help of a UV-Vis spectrophotometer [28]. For determination of proline content, about 0.5 g of fresh leaves was homogenized in sulfosalicylic acid and centrifuged. To 2 mL of the supernatant, acidic ninhydrin (2 mL) was added, and the reaction mixture was placed for one hour in a water bath at 100 °C and then cooled in an ice bath. The mixture was added with 4 mL of toluene with continuous stirring. The chromophore’s optical density containing toluene was noted at 520 nm using toluene as a blank. To determine the protein content, 0.5 g of fresh leaves was homogenized in 1 mL of phosphate buffer (7.5), and the total protein content was determined accordingly [29]. For quantifying the photosynthetic pigments, 0.5 g of leaves was homogenized in 4 mL of 80% acetone. The mixture was placed for two hours in the dark, centrifuged at 4000 rpm, and the OD of supernatant determined at 645 nm, 663 nm, and 470 nm wavelengths. Using Equations (2a–c), the contents of photosynthetic pigments were determined [30].

(1) $$\mathrm{Chl}\;a\;\left(\mathrm{mg}/g\right)=\frac{\left(12.25\times \mathrm{OD}\;\mathrm{at}\;663-2.7\times 9\mathrm{OD}\;\mathrm{at}\;645\;\right)\times \;V\;}{1000\times \;\mathrm{LW}}\;----------\;(\mathrm{Equation}\;2a)$$

(2) $$\mathrm{Chl}\;b\;\left(\mathrm{mg}/g\right)=\frac{\left(21.5\times \mathrm{OD}\;\mathrm{at}\;645-5.1\times \mathrm{OD}\;\mathrm{at}\;663\;\right)\times \;V\;}{1000\times \;\mathrm{LW}}\;----------\;(\mathrm{Equation}\;2b)$$

(3) $$\mathrm{Carotenoid}\;\left(\mathrm{mg}/g\right)=\frac{\left(1000\times \mathrm{OD}\;\mathrm{at}\;663-2.79\times \mathrm{OD}\;\mathrm{at}\;645\;\right)\times \;V\;}{1000\times \;\mathrm{LW}}\;----------\;(\mathrm{Equation}\;2c)$$

The extraction of antioxidant enzymes (EC 1.15.1.1 and EC 1.11.1.X) was accomplished by homogenizing fresh leaves (0.5 g), 0.05 N PBS (pH 7.0) containing PVPP, and 0.1 M EDTA. For determining the activity of POD (EC1.11.1.X), 0.1 mL of the supernatant of enzyme extract was added to 0.1 mL of phenylene diamine, 100 mM of MES buffer (1.35 mL and pH 5.5), and 0.05% H_2_O_2_. The change in the OD was tested at 485 nm for 3 min and the potential of POD was presented as delta OD 485 nm/min mg protein. For determining SOD (EC 1.15.1.1) activity, 0.1 mL of enzyme extract in 50 mM PBS (pH 7.8) was added to 0.1 mM EDTA, 0.075 mM NBT, 13 mM methionine, and 0.002 M riboflavin and was placed below the light chamber for 10 min, and the OD was recorded at 560 nm [31].

### 2.7. Statistical Analysis

The data were analyzed statistically using OWANOVA followed by the LSD post hoc test for the paired values comparison using statistic X software. The graphs were plotted in the OriginPro 9.1 package where each value represented the mean of triplicate data and the error bars represented 95% CL of the mean.

## 3. Results

### 3.1. Characterization of Nanoparticles

**Figure 1:**
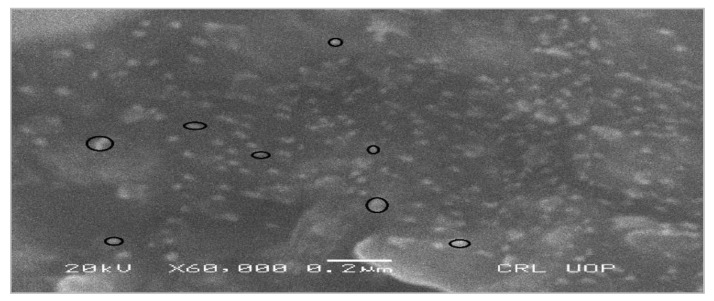
Scanning electron micrograph of Glu-FeNPs showing spherical morphology and polydisperse size distribution with size ranging from 23 to 52 nm.

**Figure 2:**
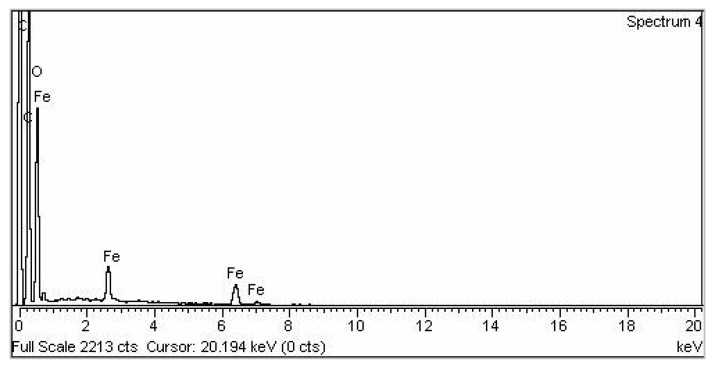
Energy-Dispersive X-ray Spectroscopy of Glu-FeNPs showing signals for elemental iron making core of FeNPs with other elements associated with surfactant.

**Figure 3:**
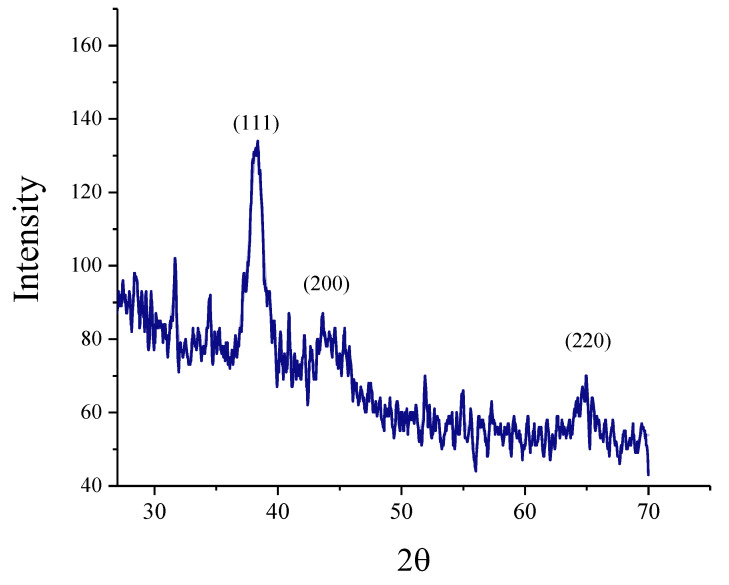
X-ray Diffraction analysis of Glu-FeNPs showing intensities at different 2θ levels corresponding to different Bragg’s planes of FCC lattice.

**Figure 4:**
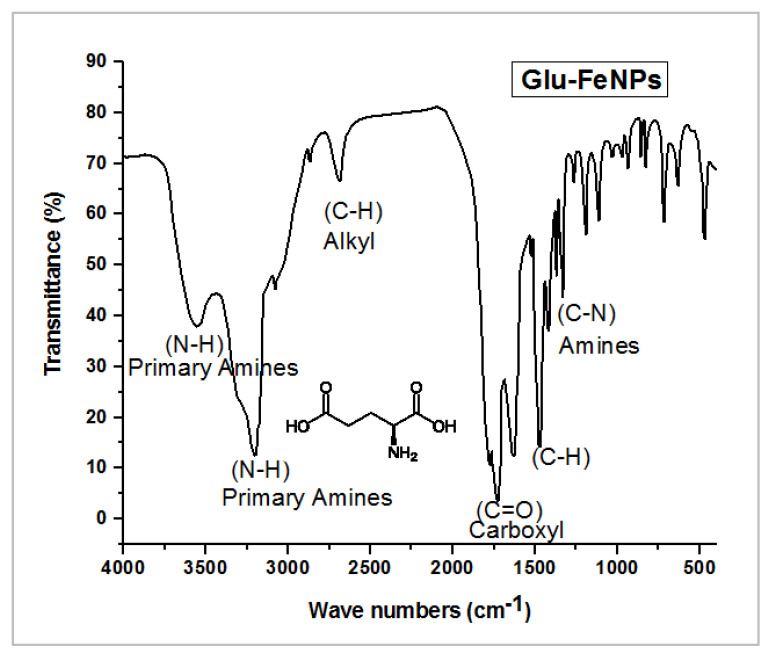
Infrared spectral analysis of Glu-FeNPs showing vibrational stretches of functional groups associated with glutamic acid, capping the FeNPs core.

Contemporary techniques were employed for determining the morphology, size distribution, crystallinity, and functional group associated with the surfactant of Glu-FeNPs. The SEM micrograph of Glu-FeNPs showed a polydispersion in size, ranging from 23 to 52 nm with a spherical morphology (Figure 1). The spot-profile EDX of iron nanoparticles revealed maximum signals of the iron at 2.1 keV due to the SPR band validating the presence of core iron in Glu-FeNPs. The presence of signals for C and O validated the presence of an organic phase in the form of glutamic acid as the surfactant (Figure 2). The crystalline nature of Glu-FeNPs was confirmed from X-ray diffraction (XRD) analysis. The XRD patterns revealed Bragg’s reflections, which are typical of iron’s fcc structure. Face-centered-cubic iron lattice characteristic diffraction peaks were at 38.1, 43.39, and 64.95 in 2θ with corresponding planes of (111), (200), and (220) (Figure 3). The Scherrer’s equation was used to calculate the average size (22 nm) of Glu-FeNPs by determining the full-width at half-maximum (FWHM) of the (111) Bragg’s reflection. Infrared spectral analysis of Glu-FeNPs showed some prominent vibrational stretches at 3560, 3208, 2690, 1731, 1630, 1473, 1422, and 1329 wavenumbers cm^−1^. The vibrational stretch at 3560 wavenumbers cm^−1^ is due to O-H bond stretching while that at 3208 wavenumbers cm^−1^ represents the presence of N-H symmetric stretching, whereas the vibrational stretch at 1731 wavenumbers cm^−1^ represents the C = O bond of carboxylic acid associated with glutamic acid (Figure 4).

### 3.2. Effect of Glu-FeNPs on Growth Performance of Mung Bean

**Figure 5:**
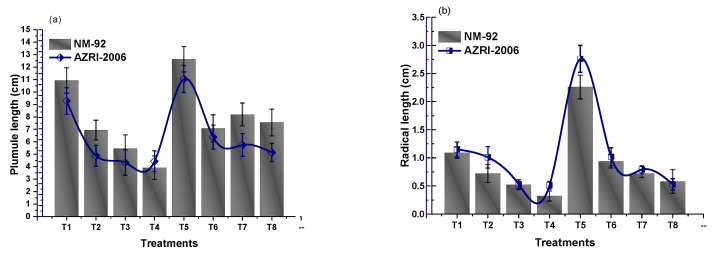
Effect of Glu-FeNPs on seedling growth performance: plumule growth (**a**) and radical growth (**b**) of two varieties (NM-92 and AZRI-2006) of *Vigna radiata* under various levels of NaCl-based osmotic stress.

**Figure 6:**
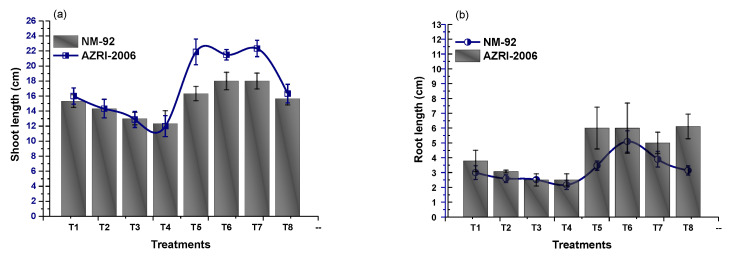
Effect of Glu-FeNPs on shoot length (**a**) and root length (**b**) of two varieties (NM-92 and AZRI-2006) of *Vigna radiata* under various levels of NaCl-based osmotic stress.

**Figure 7:**
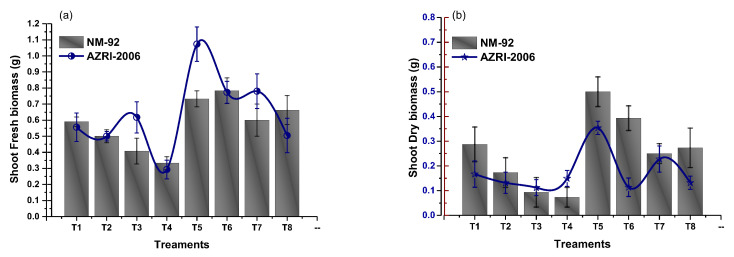
Effect of Glu-FeNPs on shoot fresh (**a**) and shoot dry biomasses (**b**) of two varieties (NM-92 and AZRI-2006) of *Vigna radiata* under various levels of NaCl-based osmotic stress.

**Figure 8:**
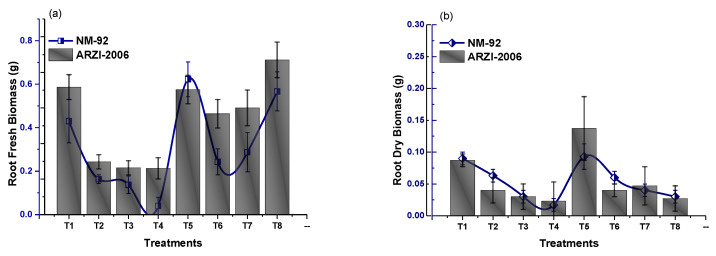
Effect of Glu-FeNPs on fresh (**a**) and dry biomasses (**b**) of root of two varieties (NM-92 and AZRI-2006) of *Vigna radiata* under various levels of NaCl-based osmotic stress.

**Figure 9:**
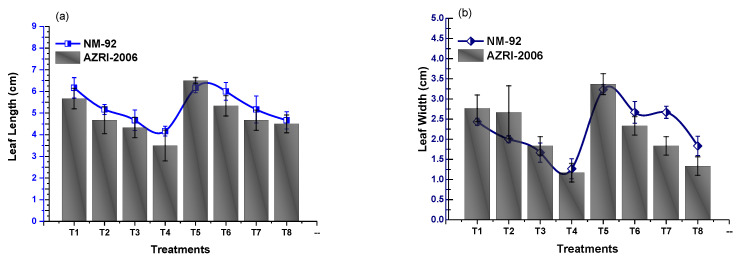
Effect of Glu-FeNPs on leaf length (**a**) and leaf width (**b**) of two varieties (NM-92 and AZRI-2006) of *Vigna radiata* under various levels of NaCl-based osmotic stress.

**Figure 10:**
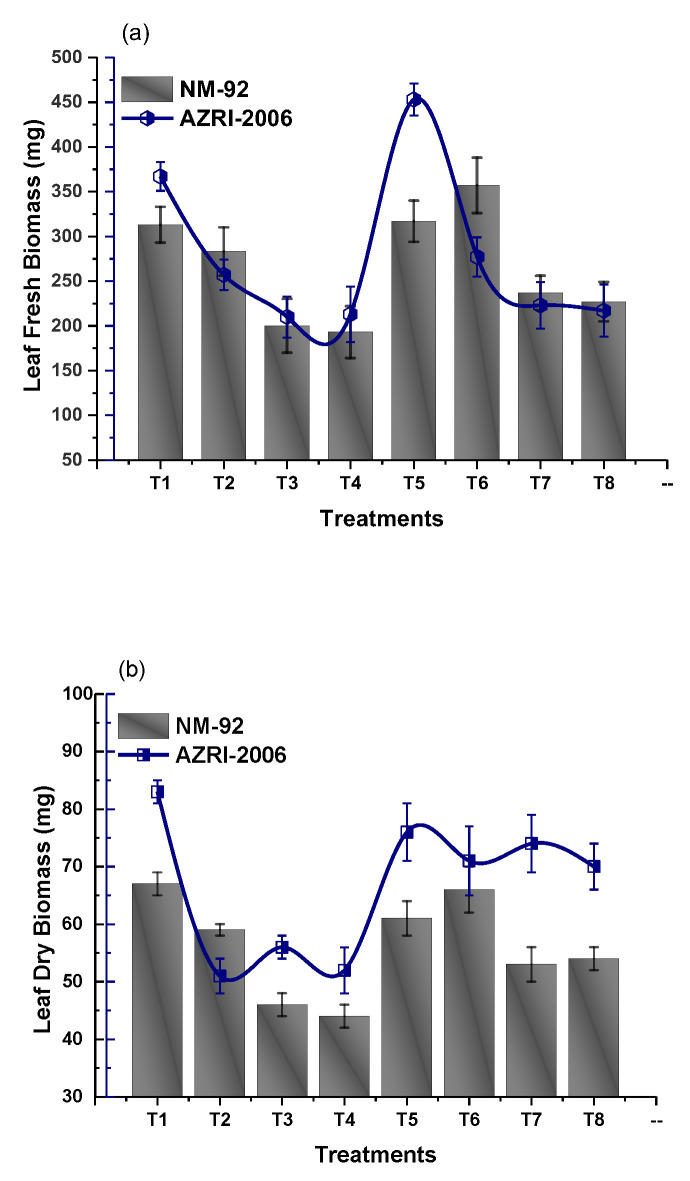
Effect of Glu-FeNPs on fresh biomass (**a**) and dry biomass (**b**) of leaves of two varieties (NM-92 and AZRI-2006) of *Vigna radiata* under various levels of NaCl-based osmotic stress.

**Figure 11:**
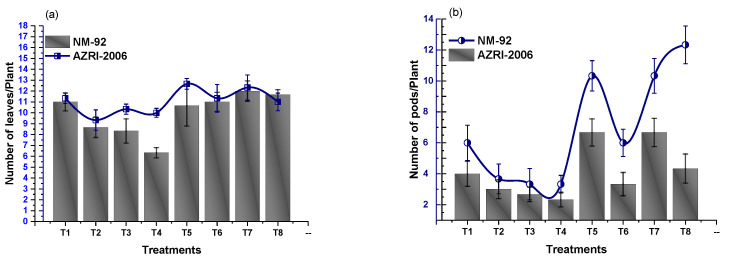
Effect of Glu-FeNPs on number of leaves/plant (**a**) and number of pods/plant (**b**) of two varieties (NM-92 and AZRI-2006) of *Vigna radiata* under various levels of NaCl-based osmotic stress.

The experimental data of the current work showed that the seeds pretreated with Glu-FeNPs significantly enhanced the growth of both varieties (NM-92 and AZARI-2006) of mung bean. The plants treated with Glu-FeNPs showed significant (*p* < 0.05) enhanced maximum growth parameters such as plumule and radical length, shoot, length, root length, dry biomass, fresh biomass, number of leaves per plant, number pods per plant, leaf length, and leaf width, as compared to the control (Table 2; Figure 5, Figure 6, Figure 7, Figure 8, Figure 9, Figure 10 and Figure 11) under the various levels of NaCl-induced osmotic stress. There was a steady reduction in all growth parameters at T2, T3, and T4, respectively, by increasing the osmotic stress (40, 60, and 80 mM NaCl) compared to T1 (control). A significant restoration in all growth parameters was observed via the application of Glu-FeNPs (T6-T8) under induced 40, 60, and 80 mM NaCl-based osmotic stress. The maximum plumule length, radicle length, and root length of 12.93 cm, 2.38 cm, and 6.11 cm, respectively, were recorded in plants whose seeds were primed with Glu-FeNPs under 80 mM NaCl-based salinity stress, while the minimum growth in the above parameter was observed (3.92 cm, 0.32 cm, and 2.5 cm, respectively) in plants without Glu-FeNPs under 80 mM NaCl-based osmotic stress (Figure 5 and Figure 6). Moreover, a considerable increase in shoot fresh weight, root fresh weight, and leaf fresh weight (1.1 g, 0.623 g, and 0.517 g, respectively) was recorded and, similarly, the dry weights of the shoot, root, and leaf (0.5 g, 0.09 g, and 0.14 g, respectively) were also observed to be higher for plants treated with Glu-FeNPs under the control (Figure 7, Figure 8, Figure 9 and Figure 10). Likewise, a minimal leaf length, leaf width, number of leaves, and number of pods per plant was recorded (4.16 cm, 1.26 cm, 6.33, and 3.33, respectively) in plants grown under the highest experimental dose (80 mM) of NaCl-based salt stress, while application of Glu-FeNPs produced a maximal leaf length (6.16 cm) and leaf width (3.23 cm) (Figure 9 and Figure 11).

### 3.3. Effect of Glu-FeNPs on Biochemical Marker

**Figure 12:**
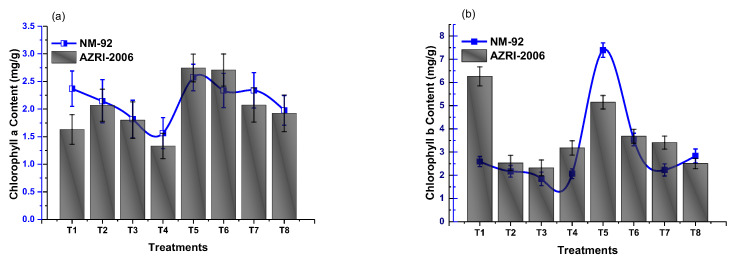
Effect of Glu-FeNPs on chlorophyll a content (**a**) and chlorophyll b content (**b**) of two varieties (NM-92 and AZRI-2006) of *Vigna radiata* under various levels of NaCl-based osmotic stress.

**Figure 13:**
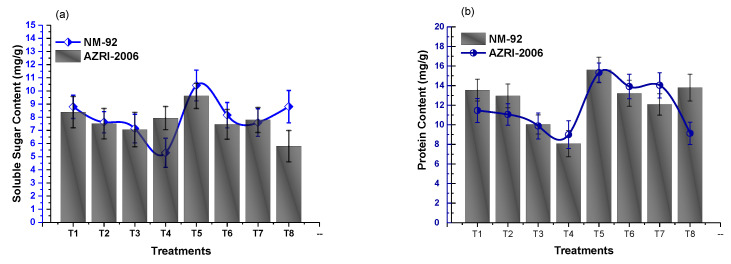
Effect of Glu-FeNPs on soluble sugar content (**a**) and protein content (**b**) of two varieties (NM-92 and AZRI-2006) of *Vigna radiata* under various levels of NaCl-based osmotic stress.

**Figure 14:**
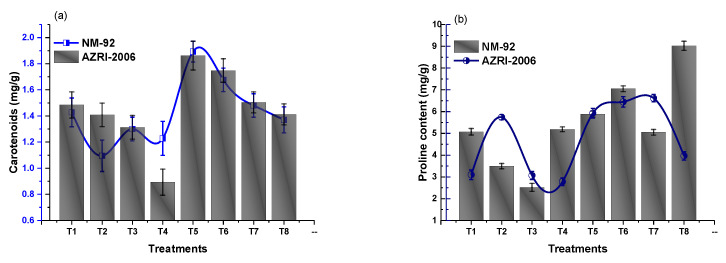
Effect of Glu-FeNPs on carotenoids (**a**) and proline (**b**) contents of two varieties (NM-92 and AZRI-2006) of *Vigna radiata* under various levels of NaCl-based osmotic stress.

The results of analysis of variance showed that chlorophyll ‘b’ and carotenoid contents significantly increased in both varieties of mung bean, treated with Glu-FeNPs in both control salinity and salt stress conditions (Table 2). The content of chlorophyll ‘a’ was non-significant statistically (*p* ≤ 0.05), but a steady decline in chlorophyll a level (1.56 and 1.33) was observed in both NM-92 and AZRI-2006 varieties, respectively, grown under 80 mM NaCl-based osmotic stress. Plants that emerged from seeds primed with Glu-FeNPs under control salinity showed maximum chlorophyll a contents (2.57 mg/g and 2.74 mg/g) in NM-92 and ARZI-2006, respectively (Figure 12). The effects of salt stress and Glu-FeNPs on chlorophyll b and carotenoid contents followed the same pattern as those of other growth parameters. Glu-FeNPs (T6, T7, and T8) mitigated the effect of osmotic stress, and the reduced chlorophylls contents in plants were restored in T6-T8 as compared to T2-T4. Maximal chlorophyll b and carotenoid (7.39 mg/g and 1.89 mg/g, respectively) contents were recorded at T5 where plants were treated with Glu-FeNPs grown under controlled osmotic stress (Figure 12 and Figure 13). Similarly, the application of Glu-FeNPs significantly increased levels of proline and protein in both varieties (NM-92 and AZRI-2006) under induced salt stress (Figure 13 and Figure 14). The increase in the level of osmotic stress (T1-T4) significantly declined sugar contents in both NM-92 and AZRI-2006 (5.29 mg/g and 7.93 mg/g, respectively), while the application of Glu-FeNPs substantially enhanced sugar (10.42 mg/g and 9.62 mg/g) in both NM-92 and AZRI-2006, respectively (Figure 14).

### 3.4. Effect on Antioxidant Enzymes

**Figure 15:**
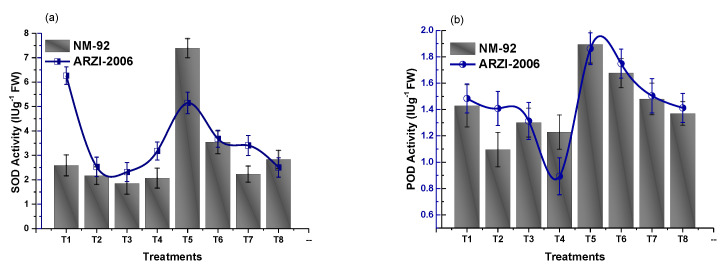
Effect of Glu-FeNPs on the activity of antioxidant enzymes, i.e., SOD (**a**) and POD (**b**), of two varieties (NM-92 and AZRI-2006) of *Vigna radiata* under various levels of NaCl-based osmotic stress.

Antioxidant enzymes (SOD and POD) are the key enzymes that play an important role in the defense system. In the present study, anti-oxidative enzyme production was studied in mung bean exposed to various doses of NaCl-based osmotic stress and under the application of Glu-FeNPs. Data depicted in Figure 15 show that osmotic stress reduced the production of SOD and POD (T2-T4), while application of Glu-FeNPs restored its production (T6-T8). It was observed that at T5 (150 mg/L of Glu-FeNPs) of AZRI-2006, a dramatic response toward the NPs was shown and the production of SOD (2.49 IU/g F.W) was enhanced to a maximum level (Figure 15). Further, the production of POD also increased (0.96 IU/g F.W and 0.92 IU/g F.W) in NM-92 and AZRI-2006, respectively, with the application of Glu-FeNPs (T5).

## 4. Discussion

The FTIR spectrum of Glu-FeNPs shows multiple variations representing various functional groups such as OH, C-H, C = H, CHO, and C-O stretching, which reveals information about Fe in Glu-FeNPs. The FTIR results are supported by the XRD and EDX profiles of Glu-FeNPs, showing an average size of 22 nm, and a spherical shape concerning Debye Scherrer’s equation and the current XRD analysis show a resemblance to previous studies of iron NPs using various plant extracts as reducing agents [21]. The pure nature of Glu-FeNPs was confirmed by EDX analysis and the presence of Fe and other elements was reported. The occurrence of carbon in the spectrum might be due to the attachment of functional groups of tannic acid, which is used as reducing agents. Our finding agrees with earlier reported research data about iron NPs [25,26].

In this experiment, all the growth parameters are affected by osmotic stress. As salinity adversely affects plant growth by reducing the osmotic potential and essential nutrients, this leads to metabolic toxicity and also increases the production of reactive oxygen species (ROS) such as superoxide (SOD) anions, hydroxyl radicals, and Na ion concentrations in different plant parts, causing toxicity [31]. The beneficial impacts of Glu-FeNPs on mung bean may be due to the coated amino acids, which has a key role in the formation of aspartic acid. This aspartic acid then metabolizes to produce amino acid lysine, threonine, methionine, and isoleucine in a series of reactions called the aspartic acid metabolic pathway. Among these, threonine has a role in the process of cell division, regulating the structure of proteins essential to cell division, and plant growth. The amino acids have the ability to be changed in polyamine molecules and regulate the movement of nitrogen among cells and organs [32]. The amino acid also acts as a precursor of spermidine and gibberellin biosynthesis, growth regulators, and many secondary metabolites [33]. It is also reported that the amino acids act as a growth regulator of cytokinin and auxin, enhancing the initiation of roots and helping with the absorption of more nutrients of the plant [34]. Another possible mechanism involved with the effect of amino acids could be due to the stimulation of root growth of Glu-FeNPs-treated plants, which may improve the water and nutrient uptake potential, enhance cell formation and cell elongation, and increase fresh and dry matter with increased growth behavior [35].

Iron, which is an important component of Glu-FeNPs, may have an important role in all growth parameters of test plants as iron is an essential microelement for plants and plays a crucial role in photosynthesis, respiration, and DNA synthesis. Its deficiency is a common disorder in many crop plants. Generally, it is present in a high quantity in soil but its availability is minimum in arable environments having neutral pH. The iron in the Fe^+3^ form cannot be utilized by plants easily; first, it is converted into a reduced form by some specialized mechanism that is difficult for plants. In our study, it may be said that Glu-FeNPs provide reduced iron for the test plant. The reduced iron is then absorbed by and transported into the roots by an iron-regulated transporter (IRT). It is reported that about 80% of elemental iron is present in those cells performing the process of photosynthesis as it is very important for nitrogen fixation, chlorophyll, the electron transport system (ETS), construction of the Fe-S cluster uptake mechanism, and other heme-enzymes [36]. These heme-enzymes such as peroxidase (SOD), different cytochromes, and catalase enzymes are present in different plant cells but their function is not known. However, some research data show that catalase enzymes are involved in the breakdown of H_2_O_2_ to harmless water and oxygen. This enzyme also has a significant role in the process of photorespiration and also improves the glycolytic cycle. The POD present in the cell wall catalyzes the reaction converting phenols to lignin. So, in iron-deficient roots, it is observed that POD activities seem to decline and the processes of cell wall formation and lignification become impaired [37]. In the chloroplast, 2–3 elemental iron atoms are present concerning PSII, 12 are in PSI, five iron atoms are related to the cytochromes complex, and ferredoxin molecules have two iron atoms. This arrangement of Fe shows that iron has the most significant role in the process of photosynthesis and yielding potential of plants.

Thus, we speculate that Glu-FeNPs is a better nano-growth-regulating agent and is also considered an efficient salt tolerance agent as it enhances the level of osmolytes and the activity of SOD enzymes in the mung bean plants and is also involved in nitrogen fixation; this may be the cause for enhancement in all growth parameters in the test plant [38,39]. He et al. [40] also found such a type of results in their experimental work and found the significant impacts of NPs on the *Citrus maxima* plant, and Rui et al. [41] reported the beneficial effects of iron NPs on the fresh and dry biomass of peanut plant under various levels of osmotic stress.

From the experimental data, it was observed that the chlorophyll and carotenoids contents decrease with increasing osmotic potential and thereafter enhance with the application of Glu-FeNPs; however, this interaction is not significant for chlorophyll ‘a’ while it is significant for chlorophyll ‘b’ and carotenoids. This decrease in the level of photosynthetic pigment in an NaCl-based osmotic stress environment leads to a decline in many biosynthetic processes or may enhance breakdown due to reactive oxygen species in chloroplast of the cell and also impair the function of the pigment protein complex. The pigments breakdown is due to the accumulation of harmful reactive ions, while the treated plants with 150 mg/L of Glu-FeNPs significantly improve the chlorophyll and carotenoids contents as compared to the control in both NaCl-based osmotic stress and osmotic-stress-free conditions. The increased chlorophylls contents enable the plant to absorb more light in photosynthesis. The results demonstrate that the applied NPs significantly enhance the growth of mung beans by improving the chlorophyll contents. The chloroplast is the main pool of Fe in plant cells, which accumulate about 80–90% of cellular iron. There is a high demand for iron in the photosynthetic apparatuses, and iron deficiency can hinder electron transporters among the two photosystems, leading to photooxidative damage [42]. In the research work, the Glu-FeNPs are considered the main supplier of glutamic acid and elemental iron as well. The Glu-FeNPs may supply the suitable quantity of Fe ions, which play a significant role in the redox reaction occurring in the cell of chloroplasts. Iron regulates the process in which the synthesis of protochlorophyllide occurs from the Mg–protoporphyrin complex and also takes part in aminolevulinic acid synthesis, which is considered the precursor of chlorophyll molecules in the biosynthesis process. Iron ions maintain a suitable apoplastic pH level and, thus, enhances the potential of Fe^3+^ reductase. The iron element is considered the most important components of many vital processes such as respiration, the electron transport chain (ETC), and photosynthesis, which help in the transportation of electrons during these processes [43]. The results of Siva and Benita [24] showed a similarity with the results of that work. In their study on the exposure of the ginger plant to iron nanoparticles, they found that iron nanoparticles significantly increased the chlorophyll and carotenoid contents.

Numerous researchers’ results confirmed that a high level of proline is beneficial in plants growing under abiotic stress conditions. Protein, sugar, and proline contents were analyzed to determine the response of the mung bean plant toward Glu-FeNPs under the various concentrations of salinity. In current research work, it was recorded that the plants exposed to Glu-FeNPs significantly increases the protein and proline contents in both varieties (NM-92 and AZRI-2006) under salt stress (Figure 13 and Figure 14). In various plants facing abiotic stresses and in water, the proline is observed as the most common and important osmolyte. Proline is important for protein protection against denaturation and scavenging reactive oxygen species (ROS), in addition to lowering the cytosolic osmotic potential. Iron acts as a carrier of glutamic acid favoring the opening of stomata and, by transamination, gives rise to the rest of the amino acids, e.g., L proline and hydroxyl proline, and this amino acid plays a key role in salt stress conditions. The study shows that Glu-FeNPs significantly enhance the level of proline as compared to that of those not treated with Glu-FeNPs. The enhancement of proline may be due to the glutamic acid present in NPs as it shows an active part in the biosynthesis of proline during various types of stress conditions [44]. The supplier of these amino acids seems to be Glu-FeNPs involved in the mechanisms to protect the plants from the adverse effects of stresses [45]. Furthermore, it is also reported that proline works as molecules-triggering agents and regulates the function of specific genes, which has an important role in scavenging free radical ions, acts as a signaling molecule triggering the expression of particular genes, and is involved in scavenging free radicals [46]. Thus, the maximum level of proline in the plant facing various concentrations of osmotic stress might be due to the application of Glu-FeNPs on the seeds of the test plant.

Antioxidant enzymes such as SOD and POD are the most important enzymes that have significant potential in the defense system of plants. Superoxide dismutase enhances the conversion of O^2-^ to H_2_O_2_ and peroxidase enzymes catalyze hydrogen peroxide [47]. At present, it was found that Glu-FeNPs significantly (*p* ≤ 0.05) enhance the tolerance level toward osmotic stress in both varieties of mung bean plants by promoting the concentration of POD and SOD enzyme activities. The response of AZRI-2006 to Glu-FeNPs in the case of SOD is maximum, while, in the case of POD, the response of NM-92 is maximum toward Glu-FeNPs. The elevated SOD and POD levels indicate that the mung bean plant strengthens its antioxidant system to deal with ROS. In various parts of plant cells such as the chloroplast, mitochondria, peroxisomes, glyoxysomes, and endoplasmic reticulum, the production of ROS occurs. To protect these organelles from the deleterious effects of ROS, various types of enzymatic and non-enzymatic genes are coded during stress conditions [48]. In the current research work, it was recorded that some genes might be activated by Glu-FeNPs, which enhance the production of antioxidant enzymes and, thus, protect the cells of plants from damage caused by oxidative stress. Overall, it could be concluded that Glu-FeNPs application under free and osmotic stress conditions enhance the potential of SOD and POD enzymes, protecting the plant from osmotic stress. These results follow the work of Das et al. [49], where the seeds of rice were treated with different doses of nano-iron pyrite. The antioxidant potential of iron NPs was observed by Rui et al. [41] and Alexandre et al. [50] using peanut and wheat plants facing various levels of osmotic stress conditions. During normal cellular activities such as respiration, photosynthesis, photorespiration, and various types of stresses, a lot of reactive oxygen species (ROS) compounds are produced. The elimination of these ROS compounds is mainly possible through the mechanism of antioxidant enzymes such as SOD and POD [51]. These enzymes are the most important enzymes that play a potentiate role in neutralizing the effect of ROS and act as a first line of defense against oxidative stress [52]. Seeds primed with iron nanoparticles may have an enhancing effect on these enzymes and thus decrease the dangerous ROS level under stress conditions. It is also reported that H_2_O_2_ levels tend to elevate in plants growing in iron-deficient soil, with the plants under such circumstances produce ROS, leading to osmotic damage. The seeds primed with iron NPs enhance the production of POD and reduce the level of H_2_O_2_.

Based on the results obtained in this experiment, we can say that Glu-FeNPs is a better growth promoter agent in mung bean, but it might be more fascinating to study the genes expression found in different parts of the cells. Furthermore, research work is required on different plants to determine the action mechanism of applied Glu-FeNPs and its possible practical application.

## 5. Conclusions

This current study reveals that the treatments of Glu-FeNPs show the most significant impact on all growth parameters of mung bean, both varieties. It was concluded that all growth parameters such as root length, shoot length, fresh and dry weight of root and shoot, number of leaves and pods per plant, moisture contents, proteins contents, contents, proline contents, chlorophylls contents, and SOD and POD activity of both varieties (NM-92 and AZRI-2006) of mung bean show a significant response toward the treatments of Glu-FeNPs under the various concentrations of NaCl-based osmotic stress. The deleterious effects of osmotic stress increase with the increase in the concentration of osmotic stresses, while the addition of Glu-NPs mitigates the deleterious effect of salinity by enhancing the potential of SOD, POD, and proline, which makes the plant resistive to osmotic stress.

## Figures and Tables

**Table 1:** Different concentrations of salinity and Glu-FeNPs given to mung bean seeds in field experiment.

Treatments	Salinity Concentration	Glu-FeNPs
T1 (Control)	Untreated	Untreated
T2	40 mM	Untreated
T3	60 Mm	Untreated
T4	80 Mm	Untreated
T5	Untreated	150 mg/L
T6	40 Mm	150 mg/L
T7	60 Mm	150 mg/L
T8	80 Mm	150 mg/L

**Table 2:** ANOVA of the effect of Glu-FeNPs on growth and physiological parameters of mung bean under NaCl-based osmotic stress.

Variables	DF	SS	MS	F	P	Sig	CI
Plumule Length	15	85.66	5.71	4.83	0.0001	***	95%
Radical	15	73.18	4.88	5.15	0.0001	***	95%
Shoot Length	15	507.91	33.86	4.64	0.0001	***	95%
Shoot Fresh Biomass	15	1.90	0.12	4.03	0.0005	***	95%
Shoot Dry Biomass	15	0.88	0.06	3.23	0.0026	**	95%
Root Length	15	92.07	6.13	2.17	0.0312	*	95%
Root Fresh Biomass	15	1.85	0.12	1.91	0.0618	NS	95%
Root Dry Biomass	15	0.052	0.003	5.05	0.0001	***	95%
No. of Leaves/Plant	15	121.91	8.12	4.24	0.0003	***	95%
Leaf Length	15	33.71	2.24	4.11	0.0003	***	95%
Leaf Width	15	19.14	1.27	8.38	0.0000	***	95%
Leaf Area	15	560.08	37.33	1.64	0.1164	NS	95%
Leaf Fresh Biomass	15	2.76	0.18	2.05	0.0417	*	95%
Leaf Dry Biomass	15	0.71	0.04	0.63	0.8270	NS	95%
No. of Pods/Plant	15	529.66	35.31	5.20	0.0000	***	95%
Protein Content	15	254.37	16.95	3.33	0.0019	**	95%
Proline Content	15	148.33	9.88	2.27	0.0243	*	95%
Total soluble Sugar	15	76.73	5.11	1.44	0.1874	NS	95%
Chlorophyll a	15	7.56	0.50	1.72	0.0956	NS	95%
Chlorophyll b	15	114.98	7.66	2.31	0.0221	*	95%
Carotenoids	15	3.14	0.20	5.11	0.0000	***	95%
SOD	15	7.02	0.46	1.29	0.2609	NS	95%
POD	15	0.96	0.06	0.32	0.9894	NS	95%

DF = Degree of Freedom, SS = Sum of squared deviation, MS = Mean deviation, F = Ratio of MSB/MSE, P = Probability, Sig = Significance, NS = Non-significant (*p* > 0.05), * = Significant (*p* < 0.05), ** = Highly significant (*p* < 0.01), *** = very highly significant (*p* < 0.001) CI = Confidence interval, POD = Peroxidase, SOD = Superoxide dismutase.

## Data Availability

Not applicable.
